# Loss of CD96 Expression as a Marker of HIV-Specific CD8^+^ T-Cell Differentiation and Dysfunction

**DOI:** 10.3389/fimmu.2021.673061

**Published:** 2021-05-27

**Authors:** Rémi Bunet, Manon Nayrac, Hardik Ramani, Mohamed Sylla, Madeleine Durand, Carl Chartrand-Lefebvre, Jean-Pierre Routy, Alan L. Landay, Jean-Francois Gauchat, Nicolas Chomont, Petronela Ancuta, Daniel E. Kaufmann, Nicole Bernard, Cécile L. Tremblay, Mohamed El-Far

**Affiliations:** ^1^ CHUM-Research Centre, Montréal, Montréal, QC, Canada; ^2^ Département de Microbiologie, Infectiologie et Immunologie, Faculté de Médecine, Université de Montréal, Montréal, QC, Canada; ^3^ Département de Médecine, Faculté de Médecine, Université de Montréal, Montréal, QC, Canada; ^4^ Research Institute of McGill University Health Centre, Montréal, QC, Canada; ^5^ Department of Internal Medicine, Rush University Medical Center, Chicago, IL, United States; ^6^ Faculté de Médecine, Département de Pharmacologie et Physiologie, Université de Montréal, Montréal, QC, Canada

**Keywords:** HIV, CD96, people living with HIV (PLWH), T-cell senescence, T-cell dysfunction, IL-32

## Abstract

Persistent immune activation and inflammation in people living with HIV (PLWH) are associated with immunosenescence, premature aging and increased risk of non-AIDS comorbidities, with the underlying mechanisms not fully understood. In this study, we show that downregulation of the T-cell immunoglobulin receptor CD96 on CD8^+^ T cells from PLWH is associated with decreased expression of the co-stimulatory receptors CD27 and CD28, higher expression of the senescence marker CD57 and accumulation of a terminally differentiated T-cell memory phenotype. In addition, we show that CD96-low CD8^+^ T-cells display lower proliferative potential compared to their CD96-high counterparts and that loss of CD96 expression by HIV-specific CD8^+^ T-cells is associated with a suboptimal response to HIV antigens. In conclusion, our results suggest that CD96 marks CD8^+^ T-cells with competent responses to HIV and the loss of its expression might be used as a biomarker for CD8^+^ T-cell senescence and dysfunction in PLWH.

## Introduction

Since the introduction of anti-retroviral therapy (ART) in 1996, mortality and morbidity associated with HIV infection have declined in people living with HIV (PLWH) (1, 2). However, as HIV persists in latent reservoirs, PLWH have to adhere to lifelong therapy to avoid viral rebound (3, 4). Furthermore, HIV persistence under ART is associated with immune activation and low-grade inflammation that drive dysregulated immune functions. The hallmark of these dysregulated functions is a state of immune exhaustion and immunosenescence (premature aging of the immune system) that are associated with the development of multiple comorbidities. At the T-cell level, two phenotypes are described: (i) T-cell exhaustion was characterized by the upregulation of multiple negative immune regulators, such as PD-1 and CTLA-4, which leads to a reversible loss of the proliferative capacity and cytokine production in response to the cognate HIV antigens; and (ii) T-cell senescence was characterized by the upregulation of surface markers, such as CD45RA, KLRG1, and CD57, with no expression of the co-stimulatory receptors CD27 and CD28 (5–7) and a loss of the proliferative capacity associated with shortened telomeres [a consequence of intense proliferative pressure in response to chronic antigenic exposure and unresolved DNA damage (8, 9)]. Although senescent cells have altered replicative capacities, they remain biologically active and may acquire a senescence associated secretory phenotypes (SASP) characterized by the production of pro-inflammatory cytokines, such as IL-6, IL-8, and IL-1β, which contribute to persistent inflammation, disruption of tissue functions and development of aging (10, 11). Yet, senescence and exhaustion phenotypes overlap in multiple characteristics that drive the T-cell dysfunction (12).

Therefore, identification of cellular proteins/receptors involved in pathways governing the development of both phenotypes in PLWH is of particular importance for T-cell potentiation (reversing T-cell exhaustion) while protecting against chronic inflammation (generation of senescent cells that may perpetuate chronic inflammation).

In this study, we investigated the role of the human CD96 molecule (also known as TACTILE: T cell-activated increased late expression) in T-cell functions and senescence. CD96 is an immunoglobulin-like receptor expressed on both NK and T-cells (13, 14). Although it was initially discovered on T-cells, much of the available knowledge on CD96 biology is related to NK cell functions (14, 15). Here, we show evidence that expression of CD96 characterizes CD8 T-cells with competent proliferation potential and function against HIV cognate antigens and the loss of which is associated with a T-cell phenotype related to dysfunction and senescence in PLWH.

## Materials and Methods

### Study Population

Peripheral blood mononuclear cells (PBMCs) used in the current study were isolated from individuals participating in three different cohorts: i) treatment naïve Elite Controllers (ECs, n=9) participating in the Canadian Cohort of HIV^+^ Slow Progressors (CCHSP), Log_10_ average viral load 1.67 ± 0.08 copies/ml and median CD4 count 842 ± 246 cells/mm^3^); ii) treatment-naïve viremic typical progressors (TP, n=9) participating in the Montreal Primary Infection (PI) cohort, Log_10_ average viral load 4.76 ± 0.51 HIV copies/ml and average CD4 count 389 ± 97 cells/mm^3^); and iii) ART-treated individuals (n=10) participating in the Canadian HIV and Aging Cohort Study (CHACS), average Log_10_ viral load 1.84 ± 0.75 HIV copies/ml and CD4 count 450 ± 292 cells/mm^3^) in addition to n=28 HIV seronegative controls.

### Ethics Statement

This study was approved by the Institutional Review Boards (IRB) of the Centre de Recherche du Centre Hospitalier de l’Université de Montréal Research (CRCHUM) and at all participating sites’ IRBs. Experiments were performed in accordance with the guidelines and regulations approved by the ethic committees from CRCHUM and all IRBs (Ethical approval #SL 04–061). Study participants provided written informed consent for use of plasma and cells in the current research investigation.

### Cells and Reagents

Peripheral blood mononuclear cells (PBMC) were isolated from blood or from leukapheresis samples by density gradient centrifugation. Cells were frozen in 90% Fetal Bovine Serum (FBS, VWR International, Radnor, PA) 10% dimethylsulfoxide (Sigma-Aldrich, St Louis, MO), and stored in liquid nitrogen until use.

### Flow Cytometry

Flow cytometry analysis was used to study the phenotype of CD96 on T-cells from HIV-positive and HIV-negative individuals using a BD LSRII FACS Analyser (BD Biosciences, San Jose, CA). Stainings was performed on 1 million of PBMCs with the following fluorochrome-conjugated antibodies: mouse anti-human CD3-Pacific Blue (Clone UCHT1), mouse anti-human CD4-Alexa Fluor 700 (Clone SK3), mouse anti-human CD8- APC-H7 (Clone SK1), mouse anti-human CD45RA-APC (Clone HI100), mouse anti-human CD27-PE-CF594 (Clone M-T271), mouse anti-human CD28-V450 (Clone CD28.2), mouse anti-human CD96-PE (Clone 6F9), and mouse anti-human CD57-FITC (Clone NK-1) (all from BD Biosciences, San Jose, CA).

### Cell Stimulations and Proliferation Assay

PBMCs were resuspended at 2 million cells/ml in RPMI 1640 medium (Gibco by Life Technologies, Waltham, MA) complemented with 10% FBS and stimulated with 500 ng/ml of IL-32α, IL-32β, or IL-32γ (R&D Systems, Minneapolis MN) in final volume of 1 ml. Cells were incubated for 5 days at 37°C and 5% CO_2_. For cell proliferation, PBMC were labeled with 2.5 μM of 5-(and-6)-carboxyfluorescein diacetate N-succinimidyl ester (CFSE) according to manufacturer’s instructions (Sigma-Aldrich, St Louis, MO) and stimulated with 1 µg/ml of phytohemagglutinin-L (PHA-L) (Sigma) and 10 ng/ml IL-2 (R&D systems) for 4 days.

### Intracellular Cytokine Staining

PBMCs were thawed and rested for 2 h in RPMI 1640 medium supplemented with 10% FBS, Penicillin-Streptomycin (Thermo Fisher scientific, Waltham, MA) and HEPES (Thermo Fisher scientific, Waltham, MA) and stimulated with 0.5 μg/ml of HIV-1 Consensus B Gag peptide pool, HIV-1 Consensus B Pol peptide pool, HIV-1 Consensus B Env peptide pool or HIV-1 Consensus B Nef peptide pool (all from JPT Berlin, Germany) corresponding to 150 15meric peptide/pool derived from Gag, Pol, Env and Nef polyproteins, respectively. Stimulations were carried out for 6 h in the presence of mouse anti-human CD107A-BV786, Brefeldin A, and monensin (BD Biosciences, San Jose, CA) at 37°C and 5% CO_2_. DMSO-treated cells served as a negative control. Cells were stained for aquavivid viability marker (Thermo Fisher scientific, Waltham, MA) for 20 min at 4°C and surface markers (30 min, 4°C), followed by intracellular detection of cytokines using the IC Fixation/Permeabilization kit (Thermo Fisher scientific, Waltham, MA) according to the manufacturer’s protocol before acquisition at a Symphony flow cytometer (BD Biosciences, San Jose, CA). The antibodies used for the surface staining were: mouse anti-human CD3-BUV395 (Clone UCHT1), mouse anti-human CD45RA-BUV563 (Clone HI100), mouse anti-human CD28-BUV737 (Clone CD28.2), mouse anti-human CD107a-BV786 (Clone H4A3), mouse anti-human CD4-BB630 (Clone SK3), CD57-FITC, CD96-PE, CD27-PE CF594, CD8-APC-H7, all from BD Biosciences, San Jose, CA. Antibodies used for the intracellular staining were the following: mouse anti-human CD69-BV650 (Clone FN50), mouse anti-human CD40L-BV711 (Clone 24–31) from Biolegend, San Diego, CA, mouse anti-human IFNg-PECy7 (Clone B27), mouse anti-human Granzym B-Alexa Fluor 700 (Clone GB11), mouse anti-human TNF-α-APC (Clone Mab11) all from BD Biosciences (San Jose, CA).

### Statistical Analysis

Data were analyzed using GraphPad Prism 8 (GraphPad Software, Inc., San Diego, CA). Comparison of two groups for the same variable were analyzed using non-parametric Mann Whitney tests (unpaired) or Wilcoxon signed-rank tests (paired comparisons). Kruskal–Wallis with Dunn’s post-tests were used to analyze more than two groups for the same variable. Between-group differences were considered statistically significant when two-tailed p-values were <0.05.

## Results

### Down-Regulation of CD96 on T Cells Is Associated With Disease Progression

**Figure 1:**
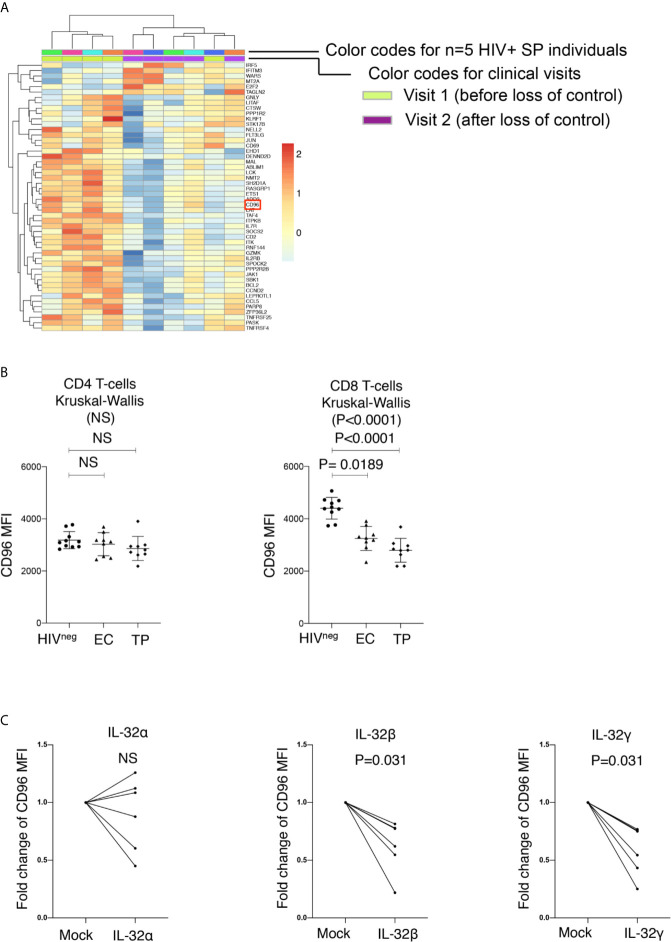
Down-Regulation of CD96 on total CD8 T-cells in HIV infection. **(A)** Heat map showing transcriptional analysis by microarrays of total PBMC from HIV^+^ slow progressor participants losing virological and immunological control (increased viral load and decreased CD4 counts, n=5) between Visit 1 (before loss of control) and Visit 2 (after loss of control). Selection of modulated genes with a cutoff of 1.3 fold and a *p* value <0.05. CD96 is highlighted by red square. **(B)** Mean fluorescence intensity (MFI) of CD96 expression assessed by flow cytometry on CD4 (left) and CD8 T-cells (right) from HIV^neg^ (n=10) as compared to Elite Controllers (EC) and Typical Progressors (TP) (n=9 *per* group). Data analyzed with the non-parametric test Kruskal-Wallis and Dunn’s subtest. **(C)** Impact of the proinflammatory cytokine IL-32 (isoforms α, β and γ) on CD96 expression on CD8 T-cells (from n=5 non-infected donors) following 5 days of stimulation. Data are shown as fold increase/decrease relative to mock-stimulated cells and analyzed with the matched pair Wilcoxon test. NS, Non-significant.

We have previously shown that persistent immune activation and chronic inflammation are associated with increased viral load and decreased CD4^+^ T-cell counts in HIV-infected slow progressors who experience loss of immunological and virological control (16). Our transcriptomic analysis on PBMCs collected from these slow progressor individuals failing control demonstrated that the human immunoglobulin-like receptor CD96 was significantly down-regulated at the transcriptional level (**Figure 1A**, full data set available at the Gene Expression Omnibus (GEO) database under the accession number GSE74790 (16)). Given the concomitant downregulation of CD96 transcription with increased viral load, we aimed to validate this phenotype at the protein level in HIV-infected typical progressors; individuals with significantly high viral loads in the absence of ART (Log_10_ average viral load 4.76 ± 0.51 HIV copies/ml) that do not meet the criteria of Slow Progressors as we previously described (16). To this end, we measured CD96 expression on CD8^+^ and CD4^+^ T-cells from ART-naive typical progressors (TP: n=9) and compared it to that in HIV-infected Elite Controllers (EC: n=9) and uninfected controls (HIV^neg^, n=10) (study participants in **Table 1**) by flow cytometry. In line with earlier reports (17), we observed that CD96 expression measured by mean fluorescence intensity (MFI) was significantly downregulated on total CD8^+^ T-cells from both EC and TP compared to HIV^neg^ individuals (p=0.0189 and p < 0.0001, respectively), although it was more pronounced in TP compared to EC. Meanwhile, differences in CD96 expression on CD4^+^ T cells in both HIV^+^ groups compared to non-infected controls did not reach statistical significance (**Figure 1B**).

**Table 1:** Demographic and clinical parameters for the study participants.

Study participants	HIV+ EC	HIV+ TP (ART-naïve)	HIV+ (On ART)	HIV^neg^
Number/group	9	9	10	28
(Men/Women)	(7/2)	(9/0)	(10/0)	(19/9)
Age (Years)	43 ± 5	40 ± 10	59 ± 6	52 ± 8*
Log_10_ Viral load	1.67 ± 0.08	4.76 ± 0.51	1.84 ± 0.75	N/A
Time since ART initiation (Years)	N/A	N/A	16 ± 6	N/A
CD4 count (Cells/mm^3^)	842 ± 246	389 ± 97	450 ± 292	NA
CD8 count (cell/mm^3^)	710 ± 281	911 ± 210	637 ± 400	NA
CD4/CD8 ratio	1 ± 0.32	0.40 ± 0.06	0.88 ± 0.62	NA

Numbers are shown in mean ± SD.

*Unknown age for n = 8 individuals.

N/A, non-applicable; NA, non-available.

CD96 expression on CD8^+^ T cells was previosuly shown to be downregulated at least in part by LPS stimulation of human PBMCs (17). Activated CD8 T-cells are known to respond to LPS *via* the surface expression of TLR4 and CD14 (18). However, the mechanism and/or the inflammatory mediator by which LPS induces CD96 downregulation is not yet clear. Here, we investigated the potential impact of IL-32, a proinflammatory cytokine that we have previously shown to be upregulated in HIV-infected slow progressors upon loss of immunoloogical and virological control and that coincides with CD96 downregulation (16). Of note, LPS is known to be a strong inducer of IL-32 (19, 20). Our data demonstrated that the dominantly expressed IL-32β and IL-32γ isoforms (21) significantly downregulated CD96 on CD8^+^ T-cells upon PBMC stimulation (**Figure 1C** p=0.031 for both). Together, these results indicated that CD96 expression on CD8^+^ T cells is subject to downregulation by IL-32, which further links this mechanism to disease progression in HIV infection.

### Loss of CD96 Expression Is Associated With a Differentiated CD8^+^ T Cell Memory and Senescence-Like Phenotypes

**Figure 2:**
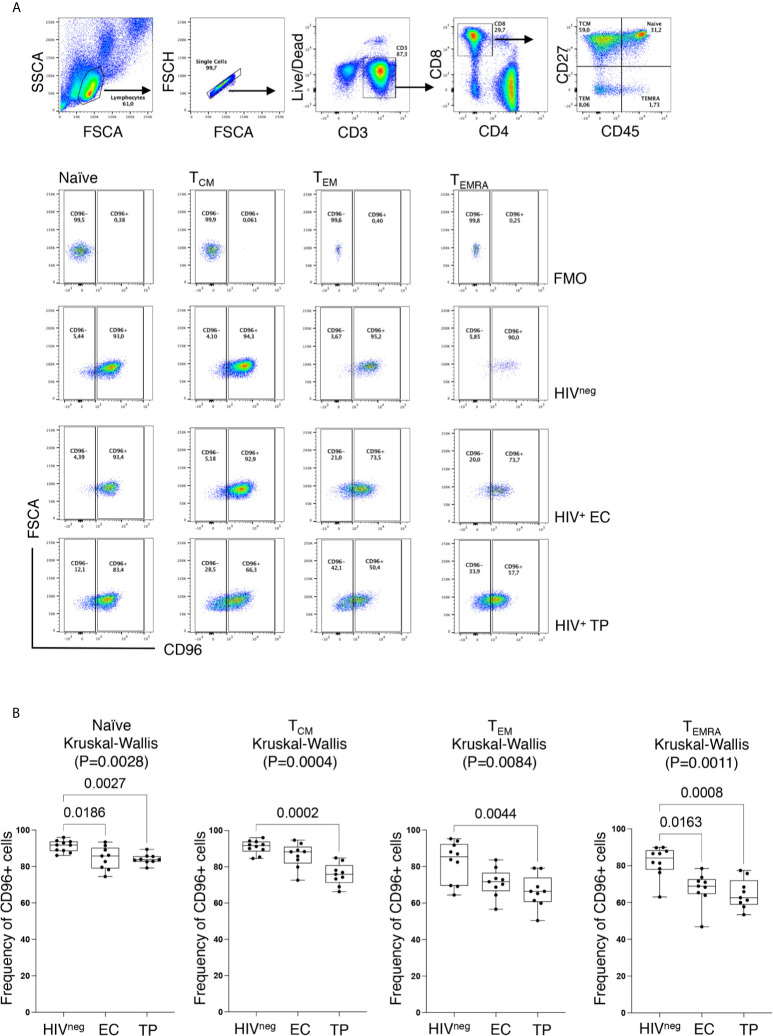
Decreased CD96 expression on CD8 T-cell subsets. **(A)** Representative *ex vivo* Flow cytometry dot plots showing the gating strategy for CD96^+^ and CD96^−^ expression on each of the four CD8 T-cell subsets; Naïve (T_N_; CD8^+^CD45RA^+^CD27^+^), Central Memory (T_CM_; CD8^+^CD45RA^neg^CD27^+^), Effector Memory (T_EM_; CD8^+^CD45RA^neg^CD27^neg^) and terminally differentiated effector memory re-expressing CD45RA (T_EMRA_; CD8^+^CD45RA^+^CD27^neg^) based on the Fluorescence Minus One (FMO) staining. **(B)** Analysis of CD96 on the different CD8 T-cell subsets from HIV^neg^ (n=10), TP (n=9) and EC (n=9) showing the decreased frequency of CD96^+^ cells in HIV^+^ individuals. Data analyzed with the non-parametric Kruskal-Wallis and Dunn’s subtest.

**Figure 3:**
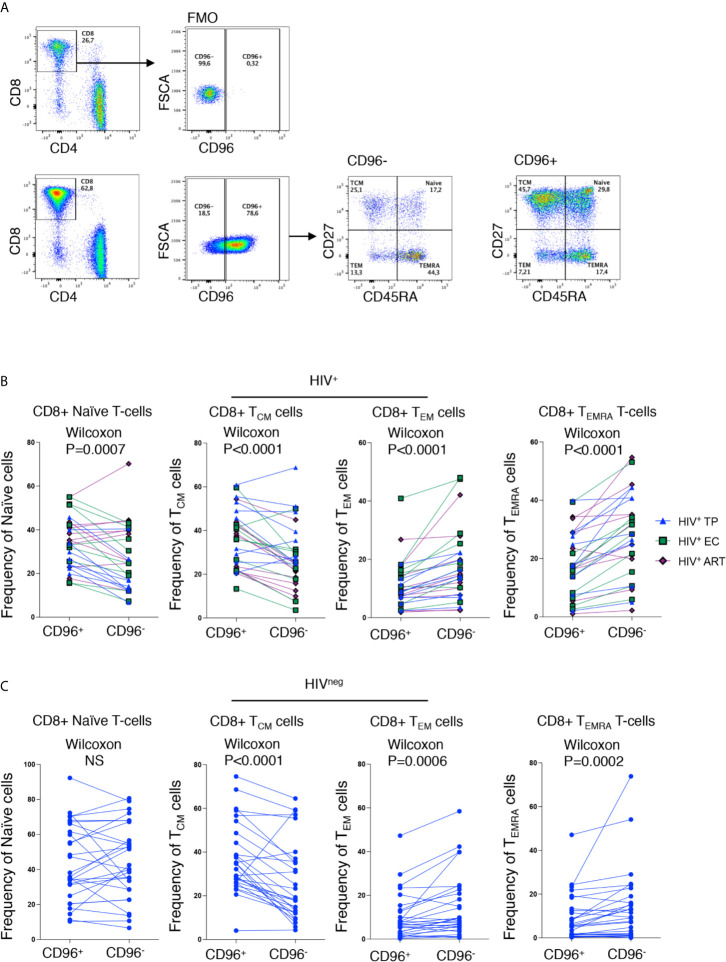
Loss of CD96 expression is associated with more differentiated phenotypes. **(A)** Representative *ex vivo* staining showing the gating strategy for CD96^+/−^ cells based on FMO (upper panels) and gating on memory subsets; T_N_, T_CM_, T_EM_ and T_EMRA_ from CD96^−^ and CD96^+^ CD8 T-cells (lower panels). **(B)** Pair analysis for the frequency of T_N_, T_CM_, T_EM_, and T_EMRA_ between CD96^−^ and CD96^+^ cells from the HIV^+^ individuals EC (n=9) TP (n=9), and ART-treated (n=10). **(C)** Same analysis as in **(B)** on HIV^neg^ (n=28). Data analyzed with Wilcoxon matched pair test. NS, Non-significant.

Similar to earlier reports (17), we observed significant loss of CD96 expression on the different CD8 T-cell subsets; Naïve (T_N_; CD8^+^CD45RA^+^CD27^+^), Central Memory (T_CM_; CD8^+^CD45RA^neg^CD27^+^), Effector Memory (T_EM_; CD8^+^CD45RA^neg^CD27^neg^) and terminally differentiated effector memory re-expressing CD45RA (T_EMRA_; CD8^+^CD45RA^+^CD27^neg^) from HIV-infected TP (**Figure 2**, p=0.0027, p=0.0002, p=0.0044, and p=0.0008, respectively). However, in the EC group, loss of CD96 expression was only significant for T_N_ and T_EMRA_ (p=0.0186 and 0.0163, respectively, **Figure 2**). To further investigate the contribution of these subsets to the pool of CD8 T-cells losing CD96 expression, we determined the frequency of T_N_, T_CM_, T_EM_, and T_EMRA_ in CD96^+^ and CD96^−^ cellscells (gated on total CD8 T-cells as shown in **Figure 3A**) from multiple groups of HIV^+^ individuals including EC, TP as well as ART-treated (**Table 1**). Interestingly, the CD8^+^CD96^−^ population was enriched with the more differentiated cells; T_EM_ (CD8^+^CD45RA^neg^CD27^neg^) and terminally differentiated effector memory (T_EMRA_; CD8^+^CD45RA^+^CD27^neg^), in the three groups of HIV^+^ individuals (**Figure 3B**, right panels, p<0.0001 for both). In contrast, the CD8^+^CD96^+^ population was enriched with the less differentiated T_N_ and T_CM_ cells from all the HIV^+^ individuals (**Figure 3B**, left panels, p=0.0007 and p<0.0001, respectively). Similar phenotypes were also observed in the HIV^neg^ controls with the exception of the T_N_ that were not different between CD96^+^ and CD96^−^ subsets (**Figure 3C**).

**Figure 4:**
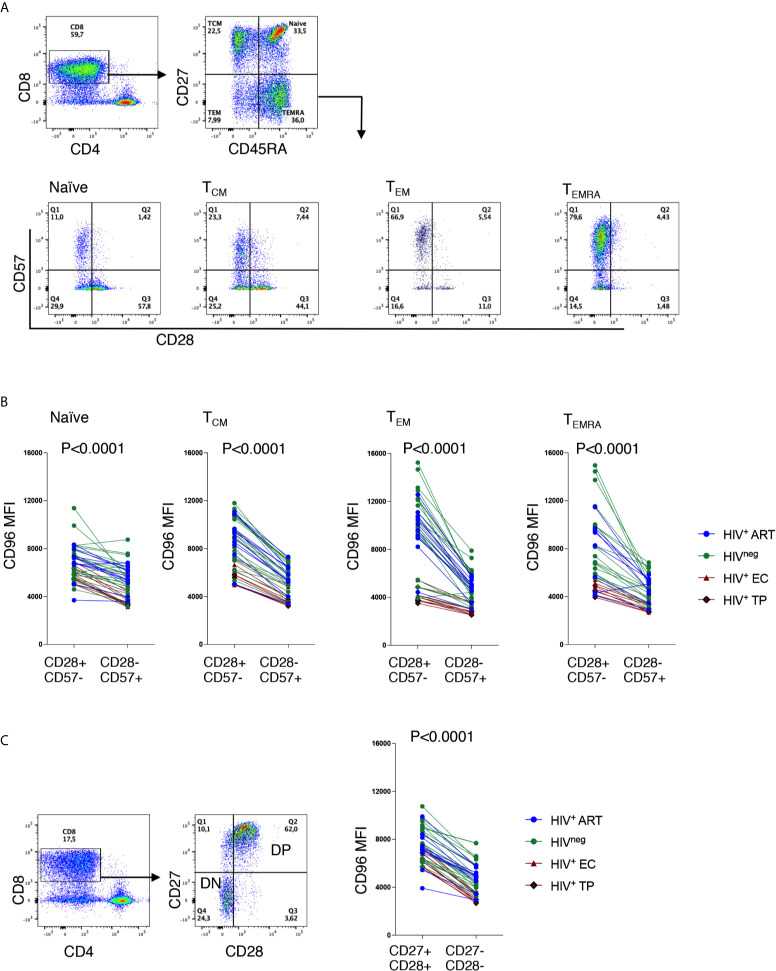
Low CD96 expression is associated with a senescence-like phenotype. **(A)** Representative *ex vivo* Flow cytometry dot plots showing the gating strategy on CD28 and CD57 from T_N_, T_CM_, T_EM_ and T_EMRA_ CD8 T-cells. **(B)** Analysis of CD96 expression on CD28^+^CD57^−^ senescent compared to their counterparts of CD28^−^CD57^+^ cells from HIV^+^ EC (n=5), TP (n=5), ART-treated (n=10) and HIV^neg^ controls (n=23). **(C)** Analysis of CD96 expression on total CD8 T-cells with CD27^+^CD28^+^ double positive (DP) cells and CD27^−^CD28^−^ double negative (DN) cells from the same individuals as in **(B)** Data analyzed with Wilcoxon matched pair test.

Given the fact that differentiated cells are enriched in CD96^−^ CD8^+^ T-cells and that the down-regulation of CD96 is associated with disease progression as shown in **Figure 1**, we hypothesized that the loss of CD96 expression might be associated with T-cell senescence. To validate this hypothesis, we assessed the expression of CD96 on CD8 T-cells with the CD28^−^CD57^+^ phenotype (a hallmark of cell senescence) within each of the four subsets; T_N_, T_CM_, T_EM_ and T_EMRA_. As shown in **Figures 4A, B**, cells with CD28^−^CD57^+^ phenotype expressed significantly lower CD96 levels, determined by the mean fluorescence intensity (MFI), when compared with CD28^+^CD57^−^ cells (p<0.0001 for all subsets). This was further confirmed by measuring CD96 on total CD8 T-cells after gating on the co-stimulatory molecules CD28 and CD27 [the loss of both is associated with cell senescence (22–24)]. As shown in **Figure 4C**, cells with the double negative DN phenotype (CD27^−^CD28^−^) expressed significantly lower levels of CD96 compared to their double positive DP counterparts (p<0.0001). We further adjusted these data for age as a confounder. ANCOVA test was run to determine the effect of different readouts (flow cytometry values) after controlling for age. Prior to linear modeling, MFI values were log2-transformed and tested for normality before testing and the effect size was reported as GES (generalized eta squared). As shown in **Table 2**, differences in CD96 expression between the different subsets remained significant suggesting these differences are age-independent. Taken together, these results demonstrate that downregulation of CD96 on CD8^+^ T-cells is associated with a senescence-like phenotype.

**Table 2:** ANCOVA analysis for adjustment of CD96 expression by age on T-cell subsets.

Population	Effect on CD96	DFn	DFd	F	P	P < 0.05	GES	P. Sig
**CD8 subsets**								
**Naïve**	CD28/CD57	1	66	17.531	8.55E-05	*	0.210	****
**T_CM_**	CD28/CD57	1	66	57.699	0.00E+00	*	0.466	****
**T_EM_**	CD28/CD57	1	66	52.672	0.00E+00	*	0.444	****
**T_EMRA_**	CD28/CD57	1	58	35.477	2.00E-07	*	0.380	****
**Total CD8**	CD27/CD28	1	66	59.856	7.84E-11	*	0.476	****

Data analysis account for comparisons of populations from the same individual.

Dfn, degrees of freedom in the numerator; Dfd, degrees of freedom in the denominator; F, statistic test for ANCOVA; P. Sig, P Significance.

*p < 0.05, ****p < 0.0001.

### Loss of CD96 Expression on CD8^+^ T Cells Is Characteristic of Low Proliferative Capacity and Poor Response to HIV Stimulation

**Figure 5:**
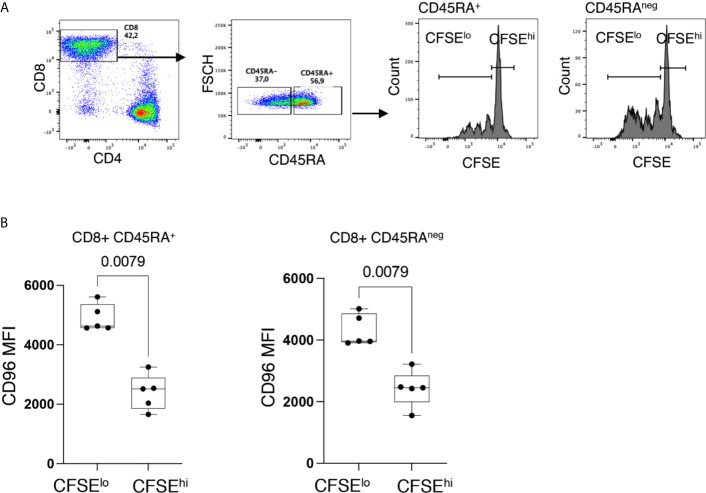
Down-regulation of CD96 is associated with poor T-cell proliferation. **(A)** Representative flow cytometry dot plots showing the gating strategy on proliferating (CFSE^lo^) and non-proliferating (CFSE^hi^) cells from CD8^+^CD45RA^+^ and CD8^+^CD45RA^neg^ T-cells. **(B)** Expression of CD96 on proliferating (CFSE^lo^) and non-proliferating (CFSE^hi^) in CD8^+^CD45RA^+^ and CD8^+^CD45RA^neg^ T-cells in response to PHA stimulation (1 μg/ml) and IL-2 (10 ng/ml) and 4 days incubation (n=5). Data analyzed with the non-parametric Mann-Whitney test.

T-cell senescence is associated with a low proliferative potential (25). To validate this hypothesis as related to CD96 downregulation, we assessed the proliferative capacity of CD96^−^ and CD96^+^ CD8^+^ T-cells, from HIV^neg^ individuals, in response to the mitogenic stimulation with PHA. Interestingly, we observed that proliferating cells (CFSE^lo^ cells) in both CD45RA^+^ and CD45RA^neg^ CD8^+^ T cells expressed significantly higher levels of CD96 compared to non-proliferating cells (CFSE^hi^ cells) (**Figure 5**, p = 0.0079 and p = 0.0079, respectively).

**Figure 6:**
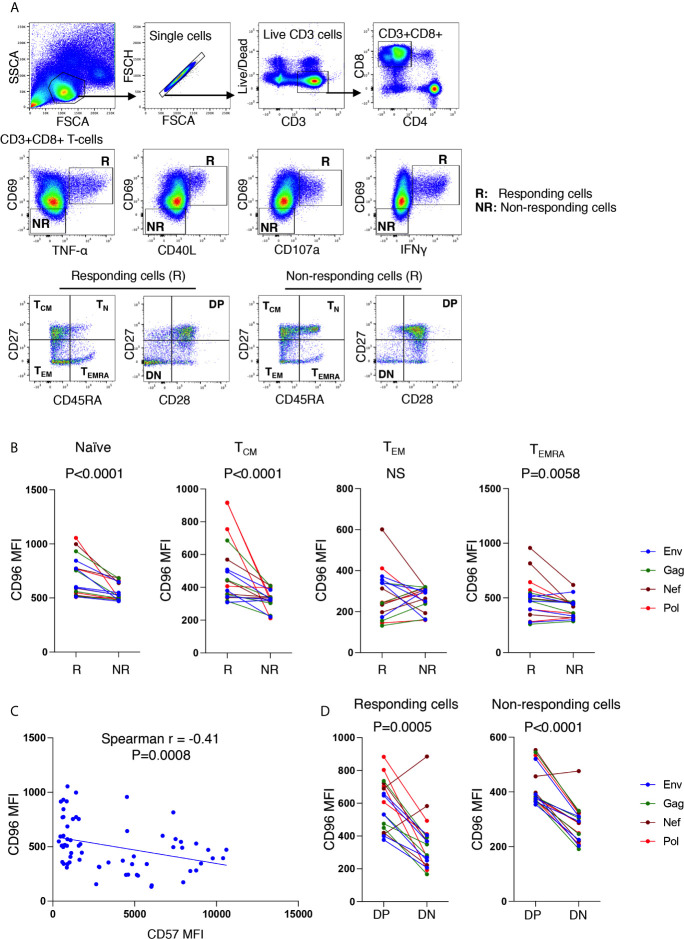
Higher expression of CD96 on responding CD8^+^ T-cells from ART-treated individuals, stimulated with HIV antigens. **(A)** Gating strategy used to identify CD3^+^CD8^+^ T-cells responding to HIV-1 peptide pools (6 h stimulation with Env, Gag, Nef, or Pol peptide pools) evaluated by intracellular cytokine staining in n=6 individuals on continuous ART (upper and middle panels). Responding cells are represented by R, whereas non-responding cells are represented by NR. Responding cells to each peptide pool (cells expression CD69 together either TNFα, CD40L, CD107, or IFNγ) were grouped together by OR Boolean gating followed by gating on the memory subsets T_CM_, T_EM_, T_EMRA_ and T_N_ as well as gating on CD27 and CD28 (lower left panels). Similar gating strategy was also used for non-responding cells (lower right panels). **(B)** Mean fluorescence intensity (MFI) of CD96 on T_CM_, T_EM_, T_EMRA_ and T_N_ from responding compared to non-responding cells (responses corresponding to cells from six participants stimulated with four peptide pools). **(C)** Correlations between CD96 MFI and CD57 MFI on all responding cells (corresponding to the pool of responses from T_CM_, T_EM_, T_EMRA_ and T_N_ from n=6 participants). **(D)** Comparison of CD96 MFI on CD27^+^CD28^+^ double positive (DP) and CD27^−^CD28^−^ double negative (DN) subsets from both responding and non-responding cells (corresponding to cells from 6 participants stimulated with 4 peptide pools). *P* values were calculated by matched-pair Wilcoxon test in **(B, D)** and Spearman correlation in **(C)**.

To further investigate this phenotype on HIV-specific T cells, we developed an intracellular staining (ICS) to identify the specific cytokine responses of the CD8^+^ T cells from ART-treated individuals (n = 6, Log_10_ average viral load 1.6 HIV copies/ml plasma and average CD4 count 590 ± 343 cells/mm^3^) to HIV-1 peptides (HIV-1 consensus B pools of Env, Gag, Nef or Pol, peptides). Following a 6-h stimulation, we characterized two cell populations: (I) activated cells that expressed one or more of the following proteins, TNF-⍺, CD40L, CD107a, and IFNγ and co-expressed the activation marker CD69 (called responding cells [R]); and (II) non-activated cells, negative for the above markers and CD69 expression [called non-responding cells [NR)] (**Figure 6A**). Interestingly, we observed that T_N_, T_CM,_ and T_EMRA_ CD8^+^ T-cells from responding cells had a significantly higher expression of CD96 compared to their counterparts of non-responding cells (**Figure 6B**, p<0.0001, p<0.0001 and p = 0.0058, respectively). However, differences in CD96 expression within the T_EM_ subsets from responding compared to non-responding cells did not reach statistical significance. Furthermore, we observed a negative correlation between the expression of CD96 and the senescence marker CD57 on the pool of responses from the different memory subsets to the 4-peptide pools (**Figure 6C**, r = −0,41, p = 0.0008). In line with these observations, CD96 expression was significantly higher on CD27^+^CD28^+^ double positive (DP) CD8^+^ T-cells when compared to the senescent-like cells with the CD27^−^CD28^−^ double negative (DN) subsets from either R or NR CD8^+^ T-cells (**Figure 6D**, p=0.0005 and p<0.0001, respectively). Taken together, these observations demonstrated that CD96 expression is associated with functionally competent responses to HIV and its loss contributes to altering the effectiveness of T-cell responses and potentially contributes to cell senescence.

## Discussion

In this study, we showed that CD96 was downregulated on CD8^+^ T-cells from HIV-infected slow progressors who experienced loss of virological and immunological control. We confirmed these observations in HIV^+^ TP, in whom CD96 downregulation was more pronounced than in EC. These data suggested that CD96 downregulation is linked to HIV disease progression and, in accordance with previous studies supporting a protective role for this protein (17). CD96 is a member of the immunoglobulin superfamily (IgSF), which is expressed on both NK and T-cells and contains an immunoreceptor tyrosine-based inhibitory motif (ITIM) (26). The presence of this ITIM suggests that CD96 may function as an inhibitory receptor. Indeed, CD96 binds to the poliovirus receptor (PVR)/CD155 to facilitate cell adhesion and also to play a positive co-stimulatory role by promoting NK redirected killing through increased adhesion to target cells (27). However, recent data, both *in vitro* and *in vivo* mouse models, showed that CD96 negatively regulates cytokine production by NK cells (15). In these studies, CD96^−/−^ mice developed NK-mediated hyperinflammatory response to toxic bacterial toxins, such as lipopolysaccharide (LPS), which led to decreased animal survival and increased death mediated by septic shock (15). These observations support the inflammatory potential of cells lacking CD96 expression. In contrast to NK cells, less is known about the exact functions of CD96 on T-cells. In line with our observations, Eriksson et al. (17), reported that CD96 expression is lower on T-cells from HIV^+^ viremic individuals and EC compared to uninfected individuals. Their studies showed that CD96^−^ CD8^+^ T-cells expressed higher levels of perforin although both CD96^+^ and CD96^neg^ cells produce similar levels of IFN-γ. These observations suggested that CD96 may play a selective or a regulatory role on the functions of some, but not all, T-cell subsets where the functions of subsets producing perforin are more regulated by CD96 expression. In line with these observations, the regulatory role of CD96 on specific T-cell functions was also supported by data from murine models where Th9 (cells producing IL-9) but not Th1 (cells producing IFN-γ) lacking CD96 expression showed highly inflammatory profiles contributing to intestinal and colonic inflammation (28). Paradoxically, an emerging role for CD96 as a negative immune checkpoint was recently proposed, since antibodies targeting CD96 seemed to enhance the anti-tumor potential of CD8^+^ T-cells in a number of mouse tumor models (29). However, it is still unclear whether this proposed role of negative regulation of T-cells by CD96 is linked with immune exhaustion, which is a typical characteristic for the expression of negative immune checkpoint receptors (30). Intriguingly, recent data on CD96 functions challenge the hypothesis of the negative regulation of T-cells (31). In these studies, antibody-mediated cross-linking of CD96 on CD8^+^ T-cells from both human and mouse clearly demonstrated that CD96 had positive co-stimulatory functions. The authors showed that CD96 triggering enhanced cytokine production and T-cell proliferation through MEK-ERK dependent pathway (31).

To gain insights into the potential role of CD96 on T-cell functions in HIV infection, we characterized the memory phenotype of cells with low compared to high expression of this immunoglobulin-like receptor. Indeed, we observed that CD96 downregulation was associated with a differentiated memory phenotype (effector memory and terminally differentiated effector memory) compared to cells with high expression of CD96 that were enriched in naïve and central memory T cell subsets. In addition, downregulation of CD96 expression was associated with loss of CD27 and CD28. In this regard, signaling provided by the co-stimulatory receptors CD27 and CD28 in the context of T-cell receptor (TCR) stimulation plays an important role in T-cell survival and IL-2 production (32, 33). However, these co-stimulatory receptors are known to be progressively downregulated as cycles of cell activation increase, consequently leading to progressive loss of the replicative capacity and accumulation of cells with the CD28^−^CD27^−^ phenotype. These later cells are compromised in their ability to up-regulate the telomerase activity (34). Such a progressive loss of surface markers is widely believed to correspond to the impairment or loss of T-cell functions (35). In addition, loss of CD27 and CD28 expression is typical of a cellular phenotype characterizing effector memory cells that re-express CD45RA (T_EMRA_), as we observed in our current studies. These cells acquire the expression of markers associated with dysfunction and senescence, such as PD-1, CD57, and KLRG-1 (36, 37).

Indeed, in our studies, we observed that cells negative for CD27 and CD28 expressed significantly lower levels of CD96, but higher levels of CD57, which links the loss of CD96 with cell senescence. This is further supported by inferior proliferative capacity of cells with lower CD96 expression in response to PHA stimulation. In addition, CD8^+^ T-cells expressing lower levels of CD96 responded poorly to HIV peptides as reflected by the absence of cytokine production compared to their counterparts with higher CD96 expression. In this regard, the absence of cytokine production by the CD96^−^ senescent-like cells is intriguing as senescent cells are known for their production of inflammatory cytokines (38, 39). These observations may suggest that loss of CD96 expression may characterize T-cells with an intermediate state preceding the senescence phase. Accumulation of such cells might contribute to the accelerated and premature aging observed in the HIV-infected individuals, which are associated with the increased burden of non-AIDS co-morbidities (40–42). This premature aging was even shown in children < 5 years old where the infection was associated with shorter telomere length (43), which is characteristic of cell senescence (44). In this regard, we acknowledge the limitation of our current study where we investigated the senescence-like phenotype of cells with low CD96 expression (cells upregulating CD57 and downregulating CD27 and CD28) and their proliferation potential without characterizing the telomere length of these cells. However, future studies are planned to address this question.

Since we observed the downregulation of CD96 in PBMCs from HIV^+^ slow progressors and a significant upregulation of the proinflammatory cytokine IL-32 in their plasma (16), it was important to study the link between these two proteins. Our data showed that IL-32 decreases CD96, consistent with earlier studies on the activation-induced downregulation of this immunoglobulin-like molecule (17). Our present study suggests that IL-32 is likely contributing to T-cell dysfunction since cells downregulating CD96 showed a poor response to challenge with HIV cognate peptides. However, it remains unclear by which mechanism IL-32 interferes with CD96 expression on CD8^+^ T-cells and further studies are needed to answer this question.

Together, our data suggest that CD96 expression is linked to T-cell differentiation and plays a protective role against loss of T-cell proliferative potential and cytokine production. The compromised expression of CD96 on T-cells by IL-32 and potentially with other inflammatory cytokines in HIV infection may lead to the accumulation of senescent-like cells with biased replicative capacities. This phenotype impairs the potential of the memory T-cells to respond to persistent HIV infection, which represents a major hurdle to viral control and a significant contributor to the development of inflammation-associated comorbidities in PLWH (45).

## Data Availability Statement

The data sets presented in this study can be found in online repositories. The names of the repository/repositories and accession number(s) can be found below: https://www.ncbi.nlm.nih.gov/geo/, GSE74790.

## Ethics Statement

The studies involving human participants were reviewed and approved by Centre Hospitalier de l’Université de Montréal Research (CHUM). The patients/participants provided their written informed consent to participate in this study.

## Author Contributions

RB planned and performed the experiments, analyzed and interpreted the data, and wrote the manuscript. MN performed the experiments on activation with HIV peptides and helped with the manuscript preparation. HR assisted with the FACS analysis. MS helped with preparation of patient samples. MD, CC-L, and J-PR provided samples from the research subjects and discussed results. AL, J-FG, and NC helped with data analysis and interpretation and revision of the manuscript. PA provided experimental layout for CFSE analysis, discussed the data, and revised the manuscript. DK helped with data analysis and interpretation, discussed the data, and revised the manuscript. NB discussed results and reviewed the manuscript. CT and ME-F planned and supervised the experiments, data analysis and interpretation and supervised the study. All authors contributed to the article and approved the submitted version.

## Funding

This work was supported by funds through the Canadian Institutes of Health Research, CIHR [grant numbers PJT 148482, HAL 398643 and PJT 168901] and National Institutes of Health, NIH [grant number R01AG054324]. This study was supported in part by the « Fonds de la Recherche Québec-Santé (FRQ-S): Réseau SIDA/Maladies infectieuses et Thérapie cellulaire ». MD is supported by a clinician researcher salary award from the Fonds de recherche en Santé- Québec. DK is an FRQS Merit Research Scholar. The CHACS is supported by the Canadian Institutes of Health Research Clinical Trial Network for HIV research.

## Conflict of Interest

The authors declare that the research was conducted in the absence of any commercial or financial relationships that could be construed as a potential conflict of interest.
